# Perspectives on physical activity participation in middle-aged U.S. South Asian women at risk for type 2 diabetes mellitus: A qualitative study

**DOI:** 10.1177/22799036261485478

**Published:** 2026-08-31

**Authors:** Nitha Mathew Joseph, Madelene Ottoson, Veronica J. Brady, Raji Pillai, Carina Katigbak, Sandy D. Jones, Larking L. Strong

**Affiliations:** 1 Cizik School of Nursing at UTHealth, 12340 The University of Texas Health Science Center, Houston, TX, USA; 2 Department of Graduate Studies, 12340 The University of Texas Health Science Center, Houston, TX, USA; 3 Department of Research, 12340 The University of Texas Health Science Center, Houston, TX, USA; 4 Andy and Barbara Gessner College of Nursing, 3992 University of Houston, Sugar Land, TX, USA; 5 Psychiatric Nurse Practitioner, Doctorate in Nursing (DNP)Student (2024), 12340 The University of Texas Health Science Center at Houston, Houston, TX, USA; 6 Department of Health Disparities Research, Division of OVP, Cancer Prevention and Population Sciences, The University of Texas MD Anderson Cancer Center, Houston, TX, USA

**Keywords:** South Asian, cardiovascular risk, diabetes, physical activity, culture, women

## Abstract

**Objective:**

Increasing evidence supports the benefits of physical activity (PA) in lowering the risk of type 2 diabetes mellitus (T2DM) among middle-aged women. Despite the increased risk for T2DM at a younger age, South Asian (SA) women in the United States (U.S.) have lower PA than men and have been studied to a lesser extent. This study explored the culturally bound attitudes, behaviors, beliefs, and barriers related to PA among middle-aged SA women at risk for T2DM.

**Design:**

A descriptive qualitative study involving focus group discussions (FGDs) with SA women (N=25) at risk for T2DM was conducted. Participants were recruited from SA religious/faith communities and professional organizations in Houston, Texas, using purposive and snowball sampling. FGDs were audio-recorded and transcribed. Data were analyzed using Braun and Clarke’s reflexive thematic analysis in MAXQDA.

**Results:**

Most participants were married, college-educated, employed, and first-generation immigrants. Two overarching themes—barriers to PA and facilitators/motivators of PA—emerged, comprising six subthemes. Barriers included balancing roles and responsibilities and cultural attitudes and norms, while facilitators/motivators included support systems, personal drives, role models, and exercise as enjoyable.

**Conclusion:**

Cultural attitudes and gender norms strongly influence PA behavior among SA women in the U.S. Collectivism expressed through bonding, accountability, enjoyment, and mutual encouragement is a key strength among SA women that shapes their PA experiences. Culturally appropriate intervention strategies may include offering peer-based, socially supported PA programs and targeted education to increase SA women’s awareness of PA benefits across life stages.

## Introduction

South Asians (SAs) (individuals from India, Pakistan, Bangladesh, Sri Lanka, Nepal, Bhutan, and the Maldives) are one of the fastest-growing Asian American subgroups in the United States (U.S.)^1^ and are at higher risk for type 2 diabetes mellitus (T2DM) than the general population.^2,3^ T2DM prevalence is higher among SAs in the U.S. (14.2%) than in African Americans (12.6%) and non-Hispanic White individuals (7.1%). Likewise, age-adjusted T2DM prevalence in SAs is 1.5 to 2 times greater than in Chinese (6.2%) or Korean (4%) subgroups in the U.S.^4^ T2DM-associated morbidity and mortality are also higher among SA immigrants than in non-Hispanic White individuals^5^ and other immigrant groups like Chinese and Japanese.^6^

Low physical activity (PA) is a notable risk factor for T2DM,^7^ and low PA levels are independently associated with T2DM development among SAs in the U.S.^8–10^ Studies found that SA immigrants engaged in low levels of PA in the US,^11^ and specifically SA women had lower levels of PA than SA men in Western country settings, including the US.^12^ In a study assessing device-measured PA among SAs in Canada,^13^ female sex and obesity (BMI ≥ 30 kg/m^2^) were significantly associated with higher sedentary time (673.5 min/day) and lower light-intensity PA (130.5 min/day), and women accumulated more sedentary time than men on average (694.6 vs 657.4 min/day; 95% CI: 671.8–718.3), demonstrating a clear sex-based disparity in PA patterns and underscoring the need for targeted health promotion in SA women.

Few studies have examined factors affecting PA in SA women in the U.S., and these were conducted in immigrant middle-aged and younger age groups.^14–16^ Nonetheless, these studies suggest that promoting PA among SA women requires careful consideration of the cultural context, as barriers to recreational PA may be culturally driven.^12,14,16^ Indeed, studies exploring social and cultural factors that influence PA behavior among SA immigrant women showed restrictive social and cultural norms discouraging exercise uptake.^12,14^ For example, SA women were less physically active than other women because of religious modesty, avoidance of mixed-gender activities, and fear of going outside alone; thus, PA remains a function relegated to routine daily duties rather than for health benefits.^12,14,17–19^ SA communities are predominantly patriarchal and collectivistic, and in this context, women often bear the responsibility of preserving and transmitting cultural traditions, values, and beliefs to their families.^20^ These cultural expectations can influence their engagement in PA.^12,18^

A deeper understanding of the social and cultural factors that contribute to PA engagement is crucial for designing and implementing effective interventions for SA women. Previous research identified immigration, language, cultural beliefs, and gender norms as common barriers to PA that operate at the individual, interpersonal, and family levels among SA women.^16^ Considering the high prevalence of T2DM in SA immigrants at younger ages than in other racial/ethnic groups, developing and evaluating effective culturally based strategies that support regular PA engagement in this high-risk population is an urgent priority.

This exploratory study aimed to bridge a critical gap in the literature by eliciting middle-aged SA women’s perspectives on acceptable forms of PA that reflect their cultural values and contextual experience as immigrants in the U.S. The purpose of this study was to examine the culturally bound attitudes, behaviors, beliefs, and barriers related to PA among middle-aged U.S. SA women at risk for T2DM.

## Methods

### Study design

A descriptive qualitative design was used. Given SA cultural preferences that value social interactions, focus groups (FGs) were considered appropriate since PA is not a sensitive subject for discussion among participants.^21^ Focus group discussions (FGDs) were conducted with middle-aged SA women at risk for T2DM to explore their perceived beliefs, benefits, motivators, and preferred methods of PA, as well as facilitators and barriers to PA. This study was reviewed and approved by the Committee for the Protection of Human Subjects at The University of Texas Health Science Center at Houston (IRB NUMBER: HSCSN-23-0139) on March 3, 2023.

### Participants and setting

Participants were recruited through purposive and snowball sampling. After obtaining permission from the community and religious leaders, the research team distributed flyers, made brief announcements, and approached women in person at Indian churches, Hindu temples, mosques, and South Asian community venues in Houston. Interested women contacted the team or provided permission to be contacted. Enrolled participants also shared study information with eligible family and peers. All potential participants who met the criteria of being SA, female, aged 40–65, English-speaking, and without T2DM or PA-limiting conditions completed the 60-second Type 2 Diabetes Risk Test (DRT).^22^ Those who scored high risk for T2DM were confirmed to be eligible and scheduled for enrollment. Individuals not at elevated risk of T2DM were excluded to ensure the sample reflected the study’s focus. The midlife period for women, defined as ages 40-65 and encompassing the late reproductive to late menopausal stages,^23^ is marked by significant hormonal, physical, and social transitions that influence women’s health behaviors. SA women in this age range face a sharply rising and disproportionately high risk of developing T2DM, often at younger ages and lower BMI thresholds than other ethnic groups,^24^ making this life stage critical for prevention. Focusing on SA women aged 40–65, therefore, captures a group at a pivotal prevention stage, where metabolic risk, caregiving responsibilities, and cultural expectations intersect to shape PA behaviors.

### Study instrument

A semi-structured FGD guide, developed by the research team, included open-ended questions, probes, and prompts based on the Theory of Planned Behavior (TPB)^25^ and is available as Supplementary File 1. The FGD guide was organized into four TPB-aligned domains: (1) attitudes toward PA, including perceived benefits and cultural meanings; (2) perceived social norms, such as family expectations, community influences, and gendered norms; (3) perceived behavioral control, addressing practical and cultural barriers, facilitators, and environmental constraints; and (4) intentions and past experiences, including motivations, prior engagement, and preferred activities. Open-ended questions with optional probes ensured consistency while encouraging participants to elaborate in depth. The guide was reviewed by content experts and piloted with a small group of participants to ensure clarity and relevance.

### Data collection

In accordance with institutional ethical regulations, all participants provided written informed consent immediately before taking part in the study. Subsequently, participants attended face-to-face 6FGDs at a pre-agreed date, time, and location as part of the data collection process. Demographic data were collected first, and FGDs were conducted using a semi-structured FGD guide (Appendix 1). A total of five FGDs were conducted, with each FGD comprising five participants and lasting 60–90 minutes. The PI, an experienced female qualitative researcher with a PhD and an associate professor at a reputable university’s nursing school (NMJ), facilitated the FGDs, while a research team member (RP) observed and took detailed field notes. Participants were informed about the FG facilitator’s role as a researcher and the purpose of the study. Several strategies were employed to minimize researcher bias during both data collection and analysis. The FG facilitator engaged in reflexive practice by writing brief memos after each FGD to document their assumptions, expectations, and positionality. This ongoing reflexive process helped maintain awareness of potential researcher influence and supported a more deliberate, critical approach to data collection and interpretations. The research team met after each FGD to reconcile reflections and potential biases. Each participant received a $25 gift card upon completion of the FGD. All FGDs were audio-recorded, with transcripts verified against audio files and field notes included to ensure data accuracy and protect against data loss. Recruitment and data collection continued until sufficient depth and breadth of data had been obtained to address the study’s aims and reach data saturation. At this point, five FGDs involving 25 participants (five per group) had been conducted. The overall study duration was one year, encompassing participant recruitment, data collection, and data analysis.

### Data analysis

Data were analyzed using Braun and Clarke’s reflexive thematic analysis^26,27^ following the six phases of familiarization, coding, theme development, review, definition, and reporting. This method was selected to support the study’s inductive approach and to identify patterns within participants’ responses. Final FGD transcripts were uploaded to MAXQDA (VERBI Software, Berlin, Germany) for data management. The analysis began with in-depth familiarization with the transcripts to gain a clear understanding of their content. Relevant excerpts were systematically extracted from the transcripts and organized into concise sentence units representing participants’ responses. These units were interpreted and coded reflexively and iteratively, with codes evolving as interpretations deepened. This process informed the development of initial themes, which were subsequently reviewed and refined through further analysis. Coded data were compared across participants to identify recurring patterns, which were organized into subthemes. These subthemes were then synthesized into overarching themes that reflected core participant perspectives. Theme development was further refined through analytic writing, team discussions, and ongoing reflexive dialogue about how researchers’ assumptions and positionalities shaped their interpretations. Final themes and subthemes were collaboratively defined, titled, and prepared for reporting. To enhance trustworthiness, the team held regular analytical meetings and reviewed reflexive journal entries to surface and bracket assumptions throughout the process.

### Trustworthiness

Extended engagement with participants (lasting 60-90 minutes per FGD) facilitated richer, more in-depth data. To ensure trustworthiness, the study adhered to Guba and Lincoln’s (1985) four criteria: credibility, dependability, transferability, and confirmability.^28^ Credibility was maintained through audio recordings, reflexive and field notes, and paraphrasing to verify participants’ responses. Dependability was supported by having team members individually validate FGD transcripts against audio recordings for accuracy, along with systematic documentation of analytic decisions. Transferability is enhanced by including verbatim quotes. Confirmability is ensured through an audit trail documenting the coding process, including how codes and patterns were identified.

### Reporting standards

The reporting of this study conforms to the COREQ (Consolidated Criteria for Reporting Qualitative Research) guidelines,^29^ and the completed checklist is provided as Supplementary File 2.

## Results

In total, 25 SA women were at high risk for T2DM. The participants had a mean age of 47.76 years (range, 40-58 years; standard deviation = 4.1) and were primarily partnered, college-educated, employed, and first-generation immigrants. Participants’ demographic characteristics are described in Table 1.

**Table 1.** Demographic data.

Total N = 25	N (%) or mean (SD)
Age (years)	47.76 (4.12)
Marital Status	
Married	92
Single	4
Divorced/Separated	4
Highest Education Completed	
College degree	​
Postgraduate/Professional degree	28
	72
Yearly Household Income	
$50,000 - $69,999	12
$70,000 - $99,999	24
$100,000 - $149,999	40
$150,000 or over	24
Employment Status	
One full-time job	92
More than one job	4
Unemployed	4
Presence of existing medical conditions	44
Age when immigrated to the U.S. (years)	28.40 (8.99)
Years lived in the U.S.	19.16 (8.49)

SD: Standard deviation.

Participants reported a wide range of PA preferences during the FGDs. Participants described enjoying (or wanting to participate in) walking during leisure time, at work, and around their neighborhoods. Participants also discussed additional preferred forms of PA, including yoga, dance, and swimming. In addition, several participants mentioned household chores, such as cleaning, cooking, and gardening, as PA. Participants working in the healthcare field described their work as a form of PA, while other participants shared that their work was predominantly sedentary, possibly reflecting the nature of work in teaching and computer technology professions.

Participants described that PA was important to them. The analysis generated two overarching themes, barriers to PA and facilitators/motivators of PA, which comprised six subthemes describing women’s perceptions of PA participation. Barriers to PA included two subthemes: (1) balancing roles and responsibilities and (2) cultural attitudes and norms. Facilitators and motivators of PA included four subthemes: (1) support systems, (2) personal drives, (3) role models, and (4) exercise as enjoyable.

### Theme 1:Barriers to PA

Participants described multiple challenges that limited their ability to engage in regular PA. This theme included two subthemes that reflected both personal and cultural influences on PA behavior, as listed below.

#### Subtheme 1.1: Balancing roles and responsibilities

Balancing roles and responsibilities influenced women’s ability to engage in PA. The participants noted a lack of time to balance many maternal and household responsibilities within their family roles, such as preparing meals, helping children with homework, transporting children to and from after-school activities, cleaning the house, and grocery shopping. Many participants also worked full-time. Some participants who worked 12-hour shifts in occupations such as nursing were often too tired from work and wanted to relax and watch TV instead of exercising. One participant described balancing roles and responsibilities as follows:

“*… I feel like I don’t have time. I have too many—kids—I mean, picking up the baby. I already work Monday through Friday. By the time I get off from work, it’s already 6:00. And then I’m like, ‘Okay, should I go pick up my son, or should I go to the gym?’ You know, it’s a battle that I always—and then, the mom guilt sets in. I’m like, ‘Oh, if I go to the gym, I have to spend an hour, and then I’m going to be late picking up my son.’ And then I’m like—the motherly hormones tell me, ‘No, you should go pick up your son and spend time with him.’ So, it’s all the battle.”* (FG4P1).

Some participants pointed out a lack of adequate social support as a barrier to balancing roles and responsibilities.

*“Because I do think my husband supports me going to the gym or working out or me losing weight. He will support that. But how much does he facilitate it? That’s a completely different story. Because if I ask him, ‘Hey, can you pick up [our] son and then come home early and feed him and put him to sleep while I work out,’ he’s not going to do that. So that is—that’s what I’m saying.”* (FG4P1).

Participating in activities for cultural engagement was important among SA women. They ensured their children and family maintained their involvement in the Indian culture, which took away time to engage in PA.

*“Weekdays, we have to go to work. We have to do something and then the weekend, that’s the time you cook, you clean, the all—everything. Everybody is occupied. Taking kids to cultural activities. Even though you are not taking rest, you are not doing the exercise. You are—I mean, from morning till the end, we don’t know what we did. I think that’s all Indian culture. Everyone stuck to the house, family, and kids. We don’t know at the end what we did. We are not even resting.”* (FG3P5).

#### Subtheme 1.2: Cultural attitudes and norms

Social norms, such as cultural attitudes and norms, significantly influenced women’s perceptions of PA. Participants described feeling fear of judgment from other women in their culture. They acknowledged that, within the Indian culture, women are concerned about body image and how others view their time spent on exercise, especially after childbirth.

*“In our culture, it is categorized, and we are conditioned from childhood in a particular way. But what I have understood as I grew up at different phases of life is that people do judge you by how you look. Your appearances matter. In our—I can speak only to Kerala, Indian culture.”* (FG1P4).

*“No, I think, especially after having a baby—so if you were—if you don’t gain weight, it’s a cultural problem. So going to the exercise is not even a thing. You cannot even imagine going to an exercise at that time. For 3 months, they say that—everything—you rest. You’re in a resting period. By the time you will gain 20, 25 pounds easily after—yeah.”* (FG4P4)

Perceptions of being “skinny” versus “fat” and “healthy” versus “beautiful” were some of the cultural barriers to PA. Concerns about body image involved judgments by others about being “too thin,” which is regarded culturally as being unhealthy.

*“No, but—I don’t want to get too skinny, because even—when I tell my husband, he’s like, ‘No.’ He doesn’t want me to get skinny, but I want to get toned. You know, there is a difference between getting fit and toned. So, I just want to make sure I get toned. That’s the only thing I want to do. But I don’t want to lose too much weight and look very skinny, unhealthy. …because back home—when you go back home, if you lose weight, they will be like, ‘Oh, you lost so much weight. Why?’ That’s another cultural thing that everyone wants you to look healthy, plump.”* (FG2P2)

Maintaining modesty prevented some participants from engaging in PA in public locations such as gyms. These participants said they did not want people from their culture to see them in exercise clothes.

*“I probably might be okay if I am with non-Asian communities, but if I’m among Asian communities, then I’m not okay doing swimming or something. It’s a psychological thing, and that sometimes prohibits me. Like if I am—I’m going to the beach, I cannot wear a swimming suit or anything on the beach, if there is a bunch of people, my own friend circle is there, or other Indian community is there.”* (FG3P4)

*“Going to exercise—I think if I see that there are a lot of Indian people going to a gym, I will not go because I see people are—because I see this, male-dominant people will look at you. I went to a gym, and I can see all of them are looking at me like an alien coming into a gym.”* (FG4P4)

Participants noted that in the patriarchal and collectivistic SA culture, recreational PA is perceived as a selfish activity, and women bear the responsibility of holding, teaching, and transmitting cultural traditions, values, and beliefs to their families

*“The mother guilt, or we always think—I think that comes from our culture. We have seen our mothers taking care of us, not caring for themselves. That’s what we have been seeing right from when we were a child, right? As a mom, it’s not you. It’s all about others, your husband or your kids. So, I think we have that mindset where you don’t—you find time for yourself and not for your family, and kind of guilt sets in. So, you try to put aside time for exercise if you have something to do for them.”* (FG4P2)

Participants also reported struggling to prioritize their health needs and goals due to the aforementioned cultural and social barriers and responsibilities.

*“So, [I’ve] been reading a lot about self-care and how to prioritize yourself over others. But again, I am not putting in enough effort to prioritize myself and my health over other things. It’s always—it’s always something that I always put at the end of my list, right?”* (FG4P1)

Of note, participants acknowledged that PA is not a learned behavior in their culture, and they had few role models, especially women, engaging in PA.

*“Also, in India, nobody goes to the gym. There was no need to exercise, so we never practiced or used to exercise. We never saw our moms or relatives or friends exercising to follow [their example].” (*FG4P2*)*

### Theme 2: Facilitators and motivators of PA

This theme reflected the factors that supported or motivated participants to engage in PA. Four subthemes are highlighted below.

#### Subtheme 2.1: Support systems

The value of having a support system was related to having people in one’s life who support their PA through encouragement, facilitation, and/or accountability. Participants described verbal support and encouragement from their spouses, family, and friends as important facilitators of PA. However, an equally important facilitator of PA was their partners’ actual support with household work, freeing up time for the women to engage in PA and promoting their accountability.

*“My family is very supportive. If I don’t go for a walk on my usual days because I have something to do, my husband will ask, ‘You’re not going for your walk today?’ And I say, ‘No, I have this to take [care of].’ [He says,] ‘Oh, no, I’ll take care of it. You go for your walk*.’” (FG4P2)

Several participants mentioned that accountability partners influenced their PA engagement. Some preferred having a partner with the same activity interest, and some preferred a friend over a family member as an accountability partner.

*“In the beginning of the month, you pay the fees and my yoga teacher is strict. If you don’t show up for several classes, she will ask you why, right? So, accountability—that makes you accountable. Yeah, these things help me keep myself on track.” (*FG2P1).

*“If my partner is there, there is no way I can skip my exercise. So, we have time for both of us—agreed time., so we don’t cheat ourselves, and we will go for that person. Also, someone consistent, because if you want to continue doing it” (FG3P5).*

*“And you have to like the same kind of activity… So, you have to find a partner who has the same interests and, hopefully, the same interests as me, so we can work out on the same days.”* (FG4P5).

*“I cannot be spending—like, on a treadmill—so boring. I don’t have anything to do with it, right? So, something to motivate you. Maybe I like a person, another woman who is like a friend that I can meet and walk [with], something like that.”* (FG4P1)

#### Subtheme 2.2: Personal drives

Personal factors, such as intrinsic motivation to engage in PA, were a critical influence for most of these SA women. They mentioned several motivating personal factors, such as knowing of a personal health benefit, witnessing PA role models within their community, and having personal priorities. One participant noted the necessity of having inner motivation:

*“I do it for myself. If I—whoever tells me—like if I don’t feel the need to do it, I won’t do it* (FG4P2)

Knowledge of the personal health benefits of PA, such as its role in disease prevention among those with a family history of cardiovascular disease or stroke, was a facilitator of PA. Although most participants emphasized cultural values equating plumpness with health, some described being more motivated to initiate exercise with the goal of losing weight for a special event, suggesting that they have internalized Western values that associate slimness with attractiveness. Mental and physical benefits of PA were also mentioned as motivators, but less often than social engagement and personal enjoyment. Weight loss and increased fitness were internal motivators for some SA women, while some discussed PA as a stress reduction method.

“*Yes, walking was a stress ventilator, because it gives relief. Also, ventilating. Yeah, it’s kind of walking along with friends and ventilating your feelings with friends.”* (FG1P4)

*“… You know, you feel good, your happy hormones are released. So you—in general, you feel good. Then, you feel like you’re contributing to your health by going to the gym. That’s one thing, and then feeling strong. So, feeling like—for me, it has always been like I have to feel strong, so it helps me to be in control of my surroundings and in the situation that I am in.” (*FG4P1*).*

Some participants’ engagement in PA was influenced by personal drives and goals, such as weight loss or looking fit and beautiful.

“You need to have something that drives you. When I started—when I was doing this, I was—I had that girl’s trip at the end of that. I wanted to fit into a certain kind of dress, and I wanted to look beautiful for my trip. So then worked hard and reached the goal.” (FG5P1).

Some participants expressed engagement in PA was influenced by personal priorities, such as having a financial commitment to a gym membership or a desire to be outdoors during nice weather.

*“I love to walk outside, more like—a park or somewhere where I can see nature and—so I’m trying now—for the past now—few months now, I’m trying to walk every day, like evening after I come back from work.”* (FG2P8*)*

#### Subtheme 2.3: Role models

Although some spoke about the importance of role models, such as other SA women in the community or their own families, others noted the absence of such role models in their lives. Participants also mentioned that community members from other ethnic backgrounds served as role models for exercise. For example, one participant predicted that by going running, she might prompt other women to say, ‘*Oh, you’re doing it. I could possibly be doing it.’* (FG4P2). Similarly, some women said that seeing an SA woman running or exercising would encourage them to do the same. However, they were motivated by seeing close friends or other people in the community being active, such as walking in the evening.

*“When you have a friend’s group, and you see one person losing weight or doing something right, or they talk about it, I think that is a big motivator when they are like, ‘Oh, you know, I did this, and this is how I eat now, and this is the changes I made, and this is what I gained from it.’ And you’re like, ‘Oh, share with me.’ And then I—I think that is also a big motivator—if you have a friend’s group that would motivate you or tell you or give you tips because it’s a journey.”* (FG5P1)

#### Subtheme 2.4: Exercise as enjoyable

Exercise enjoyment was commonly mentioned as a motivator, especially when it included a social element. Participants described dance as a culturally acceptable form of exercise that offered motivation, enjoyment, and social interaction.

“*I mean, I initially joined dance class mainly for physical activity because—of course, that will make you move some, but it’s recreation, too, because when you go there, you are with—the friends that you know—you know, the group of people that you are going there [with]…Okay, get some kind of enjoyment also, along with that physical activity.”* (FG2P2)

Participants described a preference for engaging in group-based physical activity alongside other SA women. Dancing to Bollywood videos and practicing teacher-led yoga were among their favorite activities.

## Discussion

This qualitative study explored perspectives toward PA among SA women in the U.S. at risk for T2DM, highlighting barriers and facilitators to PA engagement in this understudied group. Importantly, the findings highlight how cultural expectations, gendered responsibilities, and differing understandings of what qualifies as PA influence women’s ability and motivation to be active. While some themes align with prior research on SA women globally,^14,16,30^ this study provides new insight into how these factors manifest within the U.S., specifically among SA women at higher risk for cardiometabolic disease.

Barriers to PA cited by this sample of SA women highlight important cultural constructs that could serve as targets for future interventions. These findings are consistent with previous studies among women from other racial/ethnic minority groups (e.g., Hispanic and Black women), in which participants reported that family and household chores were more important than PA^31,32^ Since family-oriented role functions hold particular salience among SA women, future efforts to encourage PA could focus on messaging that emphasizes PA as a means to enhance one’s role as a wife, sister, and mother. Time taken away from family was perceived by participants as selfish, aligning with similar findings reported among Pakistani and Indian women.^33^ Most participants in this study noted insufficient familial support for PA, owing to the cultural expectation that females prioritize family needs over their health and well-being. Therefore, interventions aimed at increasing PA in SA women should be culturally tailored and include education of family members to increase engagement.

Weight loss was challenging for SA women due to the cultural norms in the SA community. Body size norms vary across cultures, and previous studies have shown that immigrants tend to conform to the body size norms of their host culture and the surrounding community.^34^ In one study, self-perception of body size was associated with larger body size norms among SA individuals.^34^ However, for some women, seeing differences in their bodies due to weight loss, or having others notice their weight loss, provided a sense of accomplishment and inner motivation to continue PA. Intrinsic motivation to exercise also stemmed from the knowledge of the physical and mental benefits of PA, as well as personal drives and goals. This result is consistent with the findings of another study,^14^ in which participants reported a desire to stay slim and fit to look beautiful. These results also illustrate the complex interplay between body image and cultural norms, highlighting its importance to understanding PA engagement among SA women. The influence of social and cultural factors on weight-related norms should be considered when developing personalized PA interventions among SA women.

Modesty and fear of judgment from the SA community were barriers to participating in PA in public places. In another study,^35^ women described similar concerns that discouraged PA and thus may have contributed to their reluctance to use mixed-gender facilities for PA. Our findings are consistent with a study of SA women in Scotland, which also explored cultural taboos related to exposing their bodies to members of the opposite sex.^33^ A study of second-generation British SA women also identified pressures to look “good” during PA as a barrier to PA.^36^ Female modesty in SA culture is deeply intertwined with the notion of moral judgment, which serves as an external indicator of respectability, honor, and family reputation. As such, women are expected to tend to matters of family and home. Deviating from this traditional practice to occupy public spaces—ones that are male-dominated—in which it is socially acceptable for men to enjoy leisurely time and pursuits, immediately signals that the woman is an anomaly. Those seeking to develop PA programs/interventions for SA women must consider that decisions to engage in PA may be less cognitively influenced and more socially driven (e.g., minimizing judgment).

A notable finding was the variation in how participants defined PA. Some women described household chores such as cooking, cleaning, and gardening as forms of PA, while others described PA as structured, intentional exercise. This ambiguity may contribute to an overestimation of activity levels and reduced engagement in moderate to vigorous PA. Conversely, participants in a similar study of SA women viewed PA as a structured and planned activity that occurs on a person’s own time.^37^ These stark differences in how SA women define PA encapsulate the tension that exists between strict gender roles within a collectivist culture and the individualistic orientation toward self-improvement and personal autonomy. Acknowledging that attitudes toward PA are culturally bound demands that interventions intended to encourage PA account for participants’ level of comfort and are conducted in a context that does not undermine their values.

Additionally, the study underscores the importance of culturally tailored education that distinguishes routine daily movement from health-enhancing PA. While the Western-centric view of exercise might emphasize meeting a goal, heart rate, or step count,^38^ a traditionally bound approach might involve engaging in household chores and activities with added purpose*—*that is, enhancing one’s daily routine to align with PA by adding weighted resistance or by incorporating functional movements (e.g., squats while loading the dishwasher, or countertop push-ups) to boost one’s heart rate. Thus, to engage SA women in PA, we must understand how they view PA so that recommendations are salient, resonant, and applicable. This finding is particularly relevant for SA women at risk for T2DM, as misperceptions about PA may hinder their efforts to achieve recommended activity levels. Interventions must therefore address not only behavioral barriers but also conceptual misunderstandings about what constitutes meaningful PA by including PA-related educational materials.

The significance of social support, having culturally similar role models and enjoyment during exercise were powerful facilitators of PA. These findings align with other studies^14^ demonstrating that engaging culturally matched role models can be a helpful strategy to enhance motivation and confidence in PA, particularly among women who may lack exposure and related confidence in active lifestyles within their immediate families.^36,39^ Enjoyment, particularly in the form of culturally linked activities (e.g., Bollywood dance, yoga, or group walking), facilitated PA engagement. These activities provided physical benefits and opportunities for social connection, which may enhance long-term adherence and emphasize the cultural values of collectivism expressed through bonding, shared accountability, enjoyment, and mutual encouragement. This concept of enjoyment of PA requires further investigation, as it may lead to increased commitment to PA among middle-aged SA women, and has been shown in similar studies to exert a positive effect.^39–41^ Enjoyment may be especially important for women who face significant structural and cultural barriers to PA, by sustaining their motivation despite competing demands. However, further research is needed to evaluate the effectiveness of the aforementioned culturally linked activities in improving PA among SA women.

As demonstrated in prior studies of SA women,^14,30^ intrinsic motivation also facilitated PA engagement in some of our participants. They described wanting to feel healthier, reduce stress, or achieve personal goals. Some women reported that weight loss or positive feedback from others reinforced their commitment to PA. Therefore, this study offers a nuanced explanation of PA behavior in this population, showing that intrinsic motivation, extrinsic social reinforcement, and collectivist values interact to shape engagement. This suggests a model in which culturally aligned, socially supported PA opportunities may be more effective than conventional, individual-focused PA interventions.

### Study strengths and limitations

This study has several strengths. FGDs generated rich, contextually grounded insights, and purposive and snowball sampling ensured that the data represented the experiences of a wide range of SA women within the community. Additionally, the rigorous use of the DRT^22^ in screening individuals at risk for T2DM was helpful in identifying those most in need of PA interventions. We also acknowledge that the study has some potential limitations. Participants were primarily middle-class college graduates who were employed and had high socioeconomic status, which may limit the generalizability of our study’s findings to low-income SA women in the U.S. Likewise, our findings may not fully capture the experiences of SA women with limited English proficiency or those from disproportionately represented SA subgroups.

## Future implications

The study findings highlight an opportunity to develop a peer-supported, culturally tailored PA intervention for SA women that taps into women’s social networks. PA among SA women is shaped by shared cultural values, norms, and practices—including modesty, collectivism, and strong social networks—yet existing interventions rarely leverage these strengths. Our results suggest that peer support enhances motivation for health-promoting PA among SA women, but such support remains underutilized. Participants expressed a strong desire for PA opportunities that provide socialization, accountability partners, shared goals, and supportive environments that uphold cultural expectations. Collectivism manifested through bonding, mutual encouragement, enjoyment, and group accountability emerged as a central motivator and cultural asset. These insights suggest that interventions that engage members of women’s social networks in a dyadic health-coaching model may create a supportive interpersonal context that enhances PA engagement. To ensure acceptability, feasibility, and cultural resonance, intervention components should be co-designed with members from trusted community settings, who can guide cultural tailoring, contextual relevance, and implementation strategies. A culturally sensitive intervention that integrates strategies to enhance social support, culturally familiar PA options, and problem-solving to address multilevel barriers (e.g., caregiving/childcare, transportation, gender norms, modesty concerns, limited culturally acceptable PA opportunities) has strong potential to improve PA participation and reduce cardiometabolic risk among SA women.

## Conclusions

This study emphasizes that cultural norms, family expectations, modesty concerns, and competing responsibilities strongly shape PA among SA women in the U.S. who are at risk for T2DM, while social support, culturally familiar activities, and personal motivation help facilitate their engagement. By illustrating how these factors intersect for U.S.-based SA women at elevated metabolic risk, the study provides new insight into why standard PA messages may be less effective in this population. The findings underscore the need for culturally grounded, socially supported, peer- and family-engaged approaches to improve PA participation and long-term engagement among middle-aged SA women.

## Significance for public health

Improving PA among SA women is critical for reducing cardiometabolic risk, as they face distinct cultural and social barriers to PA. Our findings show that effective interventions must account for these cultural dynamics and leverage SA communities’ strong collectivist cultural values. Bonding, shared accountability, and mutual encouragement emerged as key strengths, highlighting the central role of group cohesion, social networks, and peers in shaping PA behaviors. Culturally sensitive strategies such as women-only classes, peer-involved and socially supported community-based programs, and targeted education can align with cultural values while harnessing collective motivation. Socially supported, peer-based PA interventions offer a promising pathway to improving participation and long-term adherence among SA women.

## Supplemental material

Supplemental material - Perspectives on physical activity participation in middle-aged U.S. South Asian women at risk for type 2 diabetes mellitus: A qualitative study Supplemental material for Perspectives on physical activity participation in middle-aged U.S. South Asian women at risk for type 2 diabetes mellitus: A qualitative study by Nitha Mathew Joseph, Madelene Ottoson, Veronica J. Brady, Raji Pillai, Carina Katigbak, Sandy D. Jones, and Larking L. Strong in Journal of Public Health Research.

Supplemental material - Perspectives on physical activity participation in middle-aged U.S. South Asian women at risk for type 2 diabetes mellitus: A qualitative study Supplemental material for Perspectives on physical activity participation in middle-aged U.S. South Asian women at risk for type 2 diabetes mellitus: A qualitative study by Nitha Mathew Joseph, Madelene Ottoson, Veronica J. Brady, Raji Pillai, Carina Katigbak, Sandy D. Jones, and Larking L. Strong in Journal of Public Health Research.

## Data Availability

The datasets generated and/or analyzed during the current study are available from the corresponding author upon reasonable request.*
