# Supplementary material for: Perspectives on physical activity participation in middle-aged U.S. South Asian women at risk for type 2 diabetes mellitus: A qualitative study
Source: J Public Health Res. 2026 Aug 31;15(3):22799036261485478. doi: 10.1177/22799036261485478 (PMC13662570; doi:10.1177/22799036261485478)
Supplement: Supplementary file 1 [file sj-pdf-1-phj-10.1177_22799036261485478.pdf]

Supplementary File 1

Focus Group Interview Guide

1. Attitudes Toward Physical Activity

- How do you perceive the significance of physical activity in your life? *Probe: What makes it meaningful or not meaningful for you?*
- For you, is engaging in regular physical activity *beneficial or harmful, enjoyable or unpleasant? Probe: Can you give an example?*
- What positive or negative outcomes do you associate with exercise? *Probe: How have you experienced these outcomes?*
- How do South Asian cultural beliefs or expectations shape how you feel about being physically active? *Probe: Can you share a cultural belief that influences you?*

2. Perceived Social Norms

- How do family, friends, and community members view physical activity for women? *Probe: How does this affect your own activity?*
- What do your family and friends typically do for physical activity? *Probe: How often do you see them being active?*
- What might prevent people close to you from being active? *Probe: Can you give an example?*
- Who would approve or disapprove of you being active, and how would they show it? *Probe: Has this happened before?*
- How do South Asian cultural norms influence women's physical activity? *Probe: Are there gender expectations that affect activity?*
- How do you think your family and friends would react if you were active regularly to improve your health? *Probe: Why do you think they would react that way?*
- How much social support influences your decision to be active? *Probe: What type of support matters most?*
- Tell me about any support you've received from others. *Who supported you, and what did they do? What did you appreciate or dislike about that support?*

3. Perceived Behavioral Control

- What obstacles currently prevent you from being physically active? *(Time, childcare, safety, cultural expectations, weather, cost, access) Probe: Which of these has the biggest impact on you?*
- What factors influence your ability to be active? *Probe: Do these factors change over time?*
- What makes it easy for you to meet physical activity recommendations? *Probe: What helps you stay consistent?*
- What conditions would help you be more active? *(Time, place, women-only spaces, exercising alone vs. with others) Probe: Which condition would make the biggest difference?*
- What makes it difficult to be active, and how might those difficulties be reduced? *Probe: What support or resources would help?*

4. Intentions and Past Experiences

- Tell me about your typical physical activity habits. *What activities do you do, and where? – Who do you do them with?*
- What motivates you to engage in physical activity? *Probe: What keeps you motivated over time?*
- What past experiences have shaped how you feel about being active? *Probe: Were these experiences positive or negative?*
- What types of activities do you prefer, and why? *Probe: What makes these activities enjoyable or comfortable?*
- Do you plan to meet national physical activity recommendations next month? *If yes, how do you plan to do that? If not, what makes it challenging?*

Closing Question

Is there anything else about physical activity, your experiences, or your community that you would like to share?
