# Supplementary material for: Perspectives on physical activity participation in middle-aged U.S. South Asian women at risk for type 2 diabetes mellitus: A qualitative study
Source: J Public Health Res. 2026 Aug 31;15(3):22799036261485478. doi: 10.1177/22799036261485478 (PMC13662570; doi:10.1177/22799036261485478)
Supplement: Supplementary file 2 [file sj-pdf-2-phj-10.1177_22799036261485478.pdf]

Supplementary File 2

COREQ (COnsolidated criteria for REporting Qualitative research) Checklist

**Topic**	**Item No.**	**Guide Questions/Description**	**Reported on Page No.**
**Domain 1: Research team and reflexivity**			
Personal characteristics			
Interviewer/facilitator	1	Which author/s conducted the interview or focus group?	6
Credentials	2	What were the researcher's credentials? e.g., PhD, MD	6
Occupation	3	What was their occupation at the time of the study?	6
Gender	4	Was the researcher male or female?	6
Experience and training	5	What experience or training did the researcher have?	6
Relationship with participants			
Relationship established	6	Was a relationship established prior to study commencement?	6
Participant's knowledge of the interviewer	7	What did the participants know about the researcher? e.g. personal goals, reasons for doing the research	6
Interviewer characteristics	8	What characteristics were reported about the interviewer/facilitator? e.g., Bias, assumptions, reasons, and interests in the research topic	6
**Domain 2: Study design**			
Theoretical framework			
Methodological Orientation and Theory	9	What methodological orientation was stated to underpin the study? e.g., grounded theory, discourse analysis, ethnography, phenomenology, content analysis	7
Participant selection			
Sampling	10	How were participants selected? e.g., purposive, convenience, consecutive, snowball	5
Method of approach	11	How were participants approached? e.g., face-to-face, telephone, mail, email	5
Sample size	12	How many participants were in the study?	7
Non-participation	13	How many people refused to participate or dropped out? Reasons?	n/a
Setting			
Setting of data collection	14	Where was the data collected? e.g., home, clinic, workplace	6
Presence of non-participants	15	Was anyone else present besides the participants and researchers?	6
Description of sample	16	What are the important characteristics of the sample? e.g. demographic data, date	9
Data collection			
Interview guide	17	Were questions, prompts, or guides provided by the authors? Was it pilot	6

		tested?	
Repeat interviews	18	Were repeat interviews carried out? If yes, how many?	n/a
Audio/visual recording	19	Did the research use audio or visual recording to collect the data?	6
Field notes	20	Were field notes made during and/or after the interview or focus group?	6
Duration	21	What was the duration of the interviews or focus group?	6
Data saturation	22	Was data saturation discussed?	6
		correction?	
**Domain 3: analysis and findings**			
Data analysis			
Number of data coders	24	How many data coders coded the data?	7
Description of the coding tree	25	Did the authors provide a description of the coding tree?	n/a
Derivation of themes	26	Were themes identified in advance or derived from the data?	8
Software	27	What software, if applicable, was used to manage the data?	7
Participant checking	28	Did participants provide feedback on the findings?	n/a
Reporting			
Quotations presented	29	Were participant quotations presented to illustrate the themes/findings? Was each quotation identified? e.g. participant number	9-17
Data and findings consistent	30	Was there consistency between the data presented and the findings?	7
Clarity of major themes	31	Were major themes clearly presented in the findings?	9-17
Clarity of minor themes	32	Is there a description of diverse cases or a discussion of minor themes?	n/a

References

Tong A, Sainsbury P, Craig J. Consolidated criteria for reporting qualitative research (COREQ): A 32-item checklist for interviews and focus groups. *Int J Qual Health Care.* 2007;19(6):349-357. doi:10.1093/intqhc/mzm042
